# Increasing efficacy of primary care-based counseling for diabetes prevention: Rationale and design of the ADAPT (Avoiding Diabetes Thru Action Plan Targeting) trial

**DOI:** 10.1186/1748-5908-7-6

**Published:** 2012-01-23

**Authors:** Devin M Mann, Jenny J Lin

**Affiliations:** 1 Department of Medicine, Section of Preventive Medicine and Epidemiology, Boston University School of Medicine, Boston, MA, USA; 2 Department of Medicine, Division of General Internal Medicine, Mount Sinai School of Medicine, New York, NY, USA

## Abstract

**Background:**

Studies have shown that lifestyle behavior changes are most effective to prevent onset of diabetes in high-risk patients. Primary care providers are charged with encouraging behavior change among their patients at risk for diabetes, yet the practice environment and training in primary care often do not support effective provider counseling. The goal of this study is to develop an electronic health record-embedded tool to facilitate shared patient-provider goal setting to promote behavioral change and prevent diabetes.

**Methods:**

The ADAPT (Avoiding Diabetes Thru Action Plan Targeting) trial leverages an innovative system that integrates evidence-based interventions for behavioral change with already-existing technology to enhance primary care providers' effectiveness to counsel about lifestyle behavior changes. Using principles of behavior change theory, the multidisciplinary design team utilized in-depth interviews and *in vivo *usability testing to produce a prototype diabetes prevention counseling system embedded in the electronic health record.

**Results:**

The core element of the tool is a streamlined, shared goal-setting module within the electronic health record system. The team then conducted a series of innovative, "near-live" usability testing simulations to refine the tool and enhance workflow integration. The system also incorporates a pre-encounter survey to elicit patients' behavior-change goals to help tailor patient-provider goal setting during the clinical encounter and to encourage shared decision making. Lastly, the patients interact with a website that collects their longitudinal behavior data and allows them to visualize their progress over time and compare their progress with other study members. The finalized ADAPT system is now being piloted in a small randomized control trial of providers using the system with prediabetes patients over a six-month period.

**Conclusions:**

The ADAPT system combines the influential powers of shared goal setting and feedback, tailoring, modeling, contracting, reminders, and social comparisons to integrate evidence-based behavior-change principles into the electronic health record to maximize provider counseling efficacy during routine primary care clinical encounters. If successful, the ADAPT system may represent an adaptable and scalable technology-enabled behavior-change tool for all primary care providers.

**Trial Registration:**

ClinicalTrials.gov Identifier NCT01473654

## Introduction

Type 2 diabetes mellitus (DM2) is widespread throughout the United States, affecting 25.8 million people (8% of the US population), with an expected 48 million Americans suffering from diabetes by the year 2050 [1,2]. Effective prevention is critical to reversing this epidemic, and several studies have established that DM2 can be prevented through lifestyle behavior changes [3]. The landmark Diabetes Prevention Program (DPP) demonstrated a dramatic 58% reduction in incident DM2, with a comprehensive, resource-intensive behavioral change program for people with prediabetes [4].

Over 80% of US adults visit their primary care provider annually, creating an important venue for lifestyle-change counseling [5]. As such, the primary care provider is charged with identifying patients with prediabetes and counseling them on behavioral changes to prevent incident DM2. The literature has found mixed results of primary care counseling efforts, with some trials demonstrating modest effects in improving lifestyle behaviors such as exercise and diet and others demonstrating minimal or no effect [6,7]. Characteristics of effective lifestyle-change interventions include goal setting, physical activity prescriptions, and telephone follow-up calls as part of the behavior-change plan [8-10]. In a trial of diabetic patients in primary care, intervention participants who received a goal-setting, computer-guided, physician-led lifestyle counseling program achieved recommended physical activity levels 53% of the time compared with 26% before the intervention (*p *< .001); 32% of intervention patients lost at least six pounds compared to only 18% of controls (*p *= .006) [11].

However, traditional clinical encounters do not support effective behavior change [12]. Providers are often poorly trained about effective behavior-change techniques,[13] and the provider-patient encounter is often brief and consumed with mandatory documentation and reporting requirements. The time remaining for counseling for behavior change is therefore very short, unstructured, often ineffective, and can be a source of frustration for all parties. Physicians frequently report doubting patients' willingness to adhere to the behavioral-intervention recommendations, and this has led to only a minority of physicians spending time discussing physical activity and lifestyle changes [14,15]. These instances represent lost opportunities for health behavior-change counseling.

Health-related behavior change is grounded in multiple theoretical models that drive the current diabetes prevention efforts utilized in the DPP. Commonly used models include the Transtheoretical Model, Social Cognitive Theory, Health Belief Model, and the Self-Regulation Model [16-18]. At their core, these models rely on manipulation of patients' health cognitions such that they consciously choose healthy behaviors over unhealthy ones. Drivers for this change include perceived risk, motivation (intrinsic and extrinsic), relevance, self-efficacy, and response efficacy [17]. There is also evidence that there are many unconscious determinants of behavior as described in the Elaboration Likelihood Model (ELM) [19]. Influence or persuasion psychology incorporates both the conscious (central) and unconscious (peripheral) paths described in the ELM model to promote behavior changes. Together, all of the models suggest that there are multiple pathways to promoting behavior change that leverage both cognitive and noncognitive mechanisms. For example, cognitive-based approaches stress goal setting and feedback as potent means for promoting behavior changes, while noncognitive approaches employ social comparisons to unconsciously influence behavior [20].

**Figure 1:**
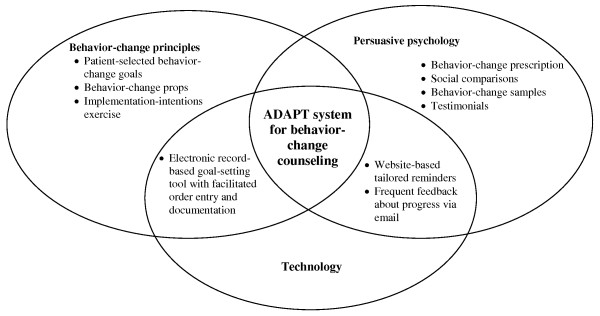
**Theoretical components of the ADAPT system**. ADAPT = Avoiding Diabetes Thru Action Plan Targeting.

Based on this rationale, we developed an innovative electronic health record (EHR)-based tool to help primary care providers rapidly and effectively counsel their prediabetes patients to improve their lifestyle behaviors. To achieve this goal, we have designed a system that incorporates elements of cognitive and noncognitive behavior-change elements adapted to the practicalities of primary care practice, including the time and cost constraints as well as the limited training of providers in behavior-change methods (see Figure 1). This paper describes the development process of this new system. The ultimate goal is to test the ability of this new system to improve physical activity, glycemia, and diet among patients with prediabetes.

## Methods/design

### Prototype development

An interdisciplinary team was assembled to begin the development process. This team included expertise from primary care, informatics, graphic design, usability, health psychology, diabetes education, and nutrition. Successful and unsuccessful models of lifestyle-change interventions in and outside of primary care were reviewed. Using an innovative combination of principles from traditional cognitive behavior change, the ELM, and principles of persuasive psychology as guides, potential behavior-change design elements for the prototype tool were selected. In addition, in-depth cognitive interviews were conducted with primary care providers and with patients with prediabetes to assess their perspectives on facilitators, barriers, and responsibilities for lifestyle-change efforts. This information was used to make final selections for the elements of the prototype tool.

### Prototype design considerations

Key design considerations for the prototype included (1) full, seamless integration within the EHR; (2) a focus on facilitating behavior changes with patients about changes they want to make rather than convincing patients that they need to make changes (*i.e*., a patient-centered, shared decision-making approach)[21]; (3) minimizing the time and cognitive load of the intervention on providers and patients; (4) having an asynchronous longitudinal component to the tool that maintains contact with patients between physician encounters; (5) simplifying the counseling process; and (6) a strong preference for evidence-based practices. The expert team also suggested incorporating "noncognitive" techniques into the intervention that could help boost the efficacy of behavioral-change counseling without additional cognitive burden on provider or patient. These included incorporation of (1) social comparisons, (2) modeling, (3) testimonials, and (4) emotional triggers. An additional objective was to create a tool that met the design specifications while minimizing the need for additional support staff to allow the intervention to be scalable for a wide range of practices.

### Initial design choices

#### Electronic health record integration

Based on the expert team review and in-depth interviews with providers and patients, a list of key elements for the study tool was generated. The first key decision was to embed the counseling tool into the commercial EHR system as provider workflow was dominated by this system and any system, regardless of quality, that disrupted this workflow would be met with resistance.

#### Prediabetes education

In addition, having providers give education on the basics of prediabetes was deemed too time consuming and generated low yield for the providers. In response, the system has intervention patients watch a 15-minute video on the basics of prediabetes developed by the American Association of Dieticians prior to their clinical encounters with their providers. Of note, this video repeatedly invokes the persuasive power of testimonials to communicate key concepts and activate the viewers to make diabetes-prevention lifestyle changes.

#### Goal setting

The goal-setting element of the DPP was considered ideal in terms of logistics and efficacy for primary care and thus chosen as a core component of the EHR intervention. Ample evidence supported the efficacy of goal setting (also known as action plans)[22], and providers in the interviews considered goal setting a feasible and reliable "widget" that they frequently used already but on an inconsistent basis. Providers also appreciated systems for integrating goal setting into their regular care with patients. There was a consensus that goal setting alone was the maximum counseling that providers could consistently incorporate into their regular practice. The goal-setting component was also designed to facilitate the development of very concrete and clear goals according to the Specific, Measurable, Attainable, Realistic, Timely (SMART) goal-setting principles [23].

#### Eliciting patient preferences in advance

**Figure 2:**
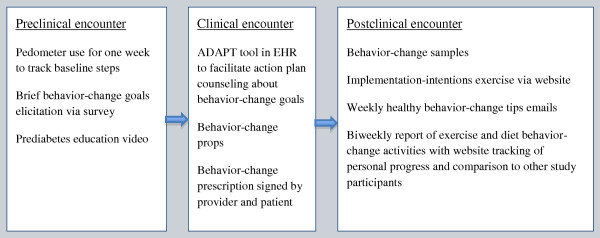
**Elements of the ADAPT system**. ADAPT = Avoiding Diabetes Thru Action Plan Targeting; EHR = electronic health record.

**Figure 3:**
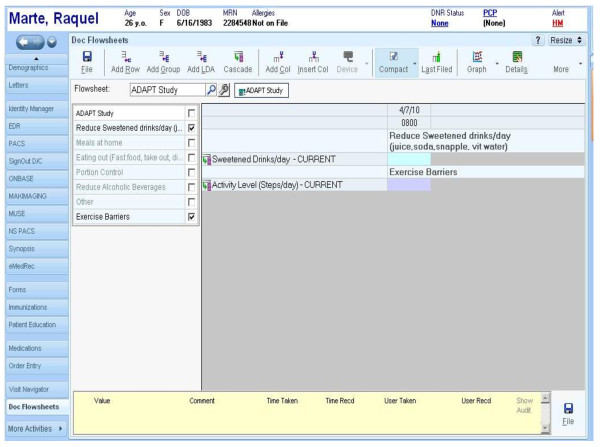
**Screenshot of test patient ADAPT goals flowsheet at a hypothetical baseline visit**. ADAPT = Avoiding Diabetes Thru Action Plan Targeting.

To facilitate the most efficacious use of provider counseling time, to incorporate patient preferences for a more shared decision-making experience, and to leverage the persuasive ability of default choices, the counseling system was designed to elicit current patient preferences for potential behavior-change goals *in advance *of the patient-provider encounter. The system also assessed patients' current levels of each of a set of prespecified common diabetogenic behaviors, such as sweetened beverage intake or consumption of fast food. This information was then presented to the provider via the EHR during the clinical encounter to facilitate counseling about changing these unhealthy behaviors (see Figure 2 for a schematic of the ADAPT system components). Many providers also noted their tendency to try to counsel on too many behavior changes at once, so the tool was designed to constrain the number of goals discussed to only one physical activity and one diet-related goal per encounter in order to lower the cognitive burden and expectations on both providers and patients (see Figure 3 for screenshot of goal-setting tool). The study was named "ADAPT - Avoiding Diabetes Thru Action Plan Targeting" to reflect the centrality of brief goal setting in the study.

#### Facilitated order entry

**Figure 4:**
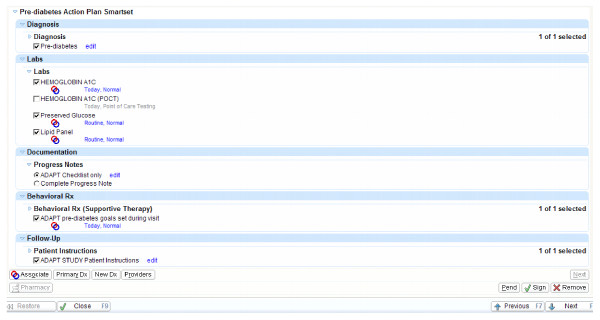
**Screenshot of ADAPT bundled order set**. ADAPT = Avoiding Diabetes Thru Action Plan Targeting.

A bundled ordering set was also linked to the counseling tool in order to facilitate rapid ordering of related tests and to allow for the creation of behavior-change prescriptions to be embedded into the visit (see Figure 4 for screenshots of the bundled order set). These behavior-change prescriptions were integrated into the patient instructions, and both patient and provider sign them in order to capitalize on the persuasive potential of written commitments [24]. This order set also auto-generated the documentation of the counseling for the visit and thus essentially eliminated the need for additional documentation of behavior counseling by the provider and potentially expedited the visit so that using the ADAPT tool for counseling could be more efficient than not using it.

#### Behavior-change props

To enhance the persuasive power of the provider interaction and help them make a more effective emotional appeal for change, providers were also provided with a tailored behavior-change prop just prior to the study encounter. For example, for patients who selected to reduce their soda intake, an empty soda bottle filled with the amount of sugar in a bottle of soda was given to providers to help them trigger an emotional reaction in their patients and help motivate behavior change (reducing number of sweetened beverages consumed). In addition, to help patients model new habits and reduce barriers to trying new behaviors, patients were given a tailored "behavior-change sample" after each visit. For example, those who chose to reduce sweetened beverages might be given samples of diet iced tea powder drinks.

#### Feedback

Feedback on progress is a key reinforcement for successful action plans [22]. To provide objective feedback on physical activity, all intervention participants are given a pedometer to track their daily activity. For their diet goal, patients are educated to self-report changes in their dietary behaviors over time. Patients and providers requested asynchronous check-in capabilities and feedback about goals between study visits. Due to the restrictions of the current EHR system, a separate website was created to prompt patients about their agreed-upon diabetes-prevention lifestyle goals on a biweekly basis. This website collected patients' self-reported data on their pedometer steps and diet goal for the previous week. To activate the persuasive power of social comparisons,[20] this information was fed back to the patients in graphical form and compared them to similar patients in the study. The data were then entered into the EHR to allow providers the ability to longitudinally monitor their patients' progress and close the feedback loop for the providers (boosting providers' self-efficacy about their behavioral-change counseling skills as well).

An additional behavior-change element of the website was an implementation-intention exercise designed to help patients visualize their action plan given two days postencounter. Implementation intentions guide participants through an "if - then" exercise that stimulates them to visualize what potential barriers to their goal may be and then to self-generate potential solutions in advance [25]. For example, if patients want to switch from regular to diet soda, the exercise would encourage them to visualize where they currently get and drink regular soda, elaborate on barriers to making the switch to diet soda (*e.g*., regular soda is what is in the machine at work), and propose their own possible solutions (*e.g*., bringing a diet soda from home to work). Implementation intentions have been shown to promote successful behavior change and are another design element of this system that do not require further effort by providers but may assist in promoting patient change [26].

### Technical considerations

#### Goal-setting interface

Early in the prototype design process, several major design issues were considered. The design team discussed several options within the EHR to house the ADAPT tool based on our prior experience with other clinical decision support tools [27], discussions with the EHR vendor, and provider workflow. We considered using a "smart" form for ADAPT because it has enhanced visual aesthetics, but it did not have the flowsheet and tracking ability we required to help providers follow their patients' behavior-change data over time. As a result, the team opted to use dynamic flowsheets for entering and tracking negotiated behavior-change goals despite its formatting limitations.

#### Restriction of alerts

The ADAPT study is a pilot, practice-based randomized clinical trial and so needed to be seamlessly integrated into workflow without disrupting control providers. As such, it was designed to activate only for providers randomized to the intervention. Furthermore, the tool is restricted to the providers' outpatient primary care EHR interface so that it does not activate during patient encounters in inappropriate settings (*e.g*., in the hospital).

#### Alert mechanism

Alerts can be categorized as interrupting versus noninterrupting and mandatory versus optional. Prior literature has demonstrated the superior efficacy of active mandatory alerts; however, they are more disruptive to workflow, which contributes to the low uptake of clinical decision support tools [28]. As patients with prediabetes often have other comorbidities and it is a chronic disease, making the ADAPT tool alert active and mandatory was viewed as too disruptive to clinical practice. We chose instead to make the ADAPT tool alert noninterrupting and optional so that the provider would need to actively choose to use the tool during an encounter. This design puts the burden on the designers to make the tool value adding enough to incentivize providers to use it rather than mandate its use. Furthermore, since prediabetes is just one of many competing priorities in a routine primary care encounter, being more proscriptive with providers about how to conduct a patient encounter is unrealistic and is likely what contributes to low rates of utilization of mandated clinical decision support tools [29].

#### Usability

Usability testing was conducted to evaluate the prototype's ability to facilitate provider counseling. To enhance the potential "real-world" application of the tool in clinical practice, *in vivo *practical usability was selected over traditional, artificially controlled testing environments [30]. Using a previously developed methodology [27], usability testing was conducted in two distinct phases. In phase one, a think-aloud protocol was used with seven primary care providers who followed a scripted interaction with a hypothetical patient while using the prototype tool. This interaction was facilitated by a study team member who sat next to the study provider to troubleshoot problems, observe the interaction, and probe the user if needed. The encounter was audio-recorded and screen capture software (Hypercam, Hyperionics Technology, LLC, Murrysville, PA, USA) was used to observe interactions with the tool. Encounters were conducted until data saturation, where no new significant themes or observations emerged, was achieved. All of the observations were then coded and used to revise the prototype. Phase two of the usability testing enhanced the "real-world" simulation by having a set of six different study providers use the prototype tool to counsel "near-live" simulated patient encounters (trained study staff served as simulated patients) in their usual clinic rooms. These data were coded and used to perform a second round of tool revisions, which led to the finalized study tool.

#### Final design

Using all of the feedback generated from the interviews and usability testing, a final ADAPT prediabetes counseling tool was generated and moved into the production version of a commercial EHR system (see Figures 3 and 4). In addition, pre-encounter brief survey questionnaires to elicit patient preferences for action plans and a behavior-tracking and feedback website, with implementation-intentions exercises, were created. Figure 2 displays how a patient flows through the ADAPT system.

### Trial design

#### Practice setting

The study is being conducted at a large, urban academic medical center. All of the providers were members of the academic primary care practice that is located on the main hospital campus. The outpatient clinic has over 55,000 visits annually and serves a diverse population that is approximately 56% Hispanic, 35% African American, 7% white, and 2% other.

#### Provider eligibility, consent, and randomization

**Figure 5:**
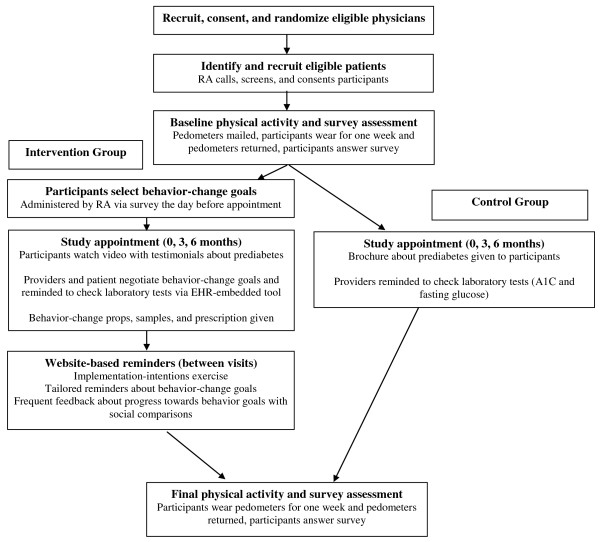
**Flow design for ADAPT randomized controlled trial**. ADAPT = Avoiding Diabetes Thru Action Plan Targeting; RA = research assistant; EHR = electronic health record.

All attending-level primary care providers who have seen at least 10 patients with prediabetes in the prior year in the medical practice are eligible for the study. The study design is a randomized control trial (RCT) in which the providers within the academic medical center outpatient practice are the unit of randomization. Faculty providers are randomized via random number generator to intervention or control in a 1:1 ratio. Only providers randomized to the intervention are triggered by the EHR to use the ADAPT tool when enrolled patients arrive for a visit. After randomization, all intervention-group providers are invited to standardized educational forums for consent and training on use of the EHR-embedded ADAPT tool (see Figure 5 for a diagram of the study flow). Randomization began in November 2011, with 20 primary care providers being enrolled. Patient recruitment is ongoing with an expected completion date of approximately one year.

#### Provider training

All providers allocated to the intervention receive approximately 45 minutes of training on how to use the ADAPT tool and what the ADAPT study experience will be from their patients' perspectives. Each training session is led by at least one study investigator and one study staff member. The training consists of a background on brief action planning, several walkthroughs of the ADAPT tool using the EHR training version, screenshots of the patient goal-elicitation survey, screenshots of the prediabetes video and website, and a demonstration video simulating use of the ADAPT tool in a live clinical encounter.

#### Patient consent

Once providers are enrolled, the EHR is queried to identify members of their panel with prediabetes. These patients are then contacted via telephone and invited to participate. A consent form is mailed home and discussed via a second phone call and verbal consent is obtained. A baseline survey is conducted on all patients (see Outcome section for specific domains). All patients are then mailed a pedometer (Omron HJ-170) to wear for one week and instructed to mail it back to the research team. A regular visit with their primary care provider was then scheduled for up to one month after the pedometer data were collected. On the night before the regular clinical visit, all patients were called to remind them of the visit, and the brief behavior-change goals-elicitation questionnaire was administered to intervention patients.

#### Trial flow

**Figure 6:**
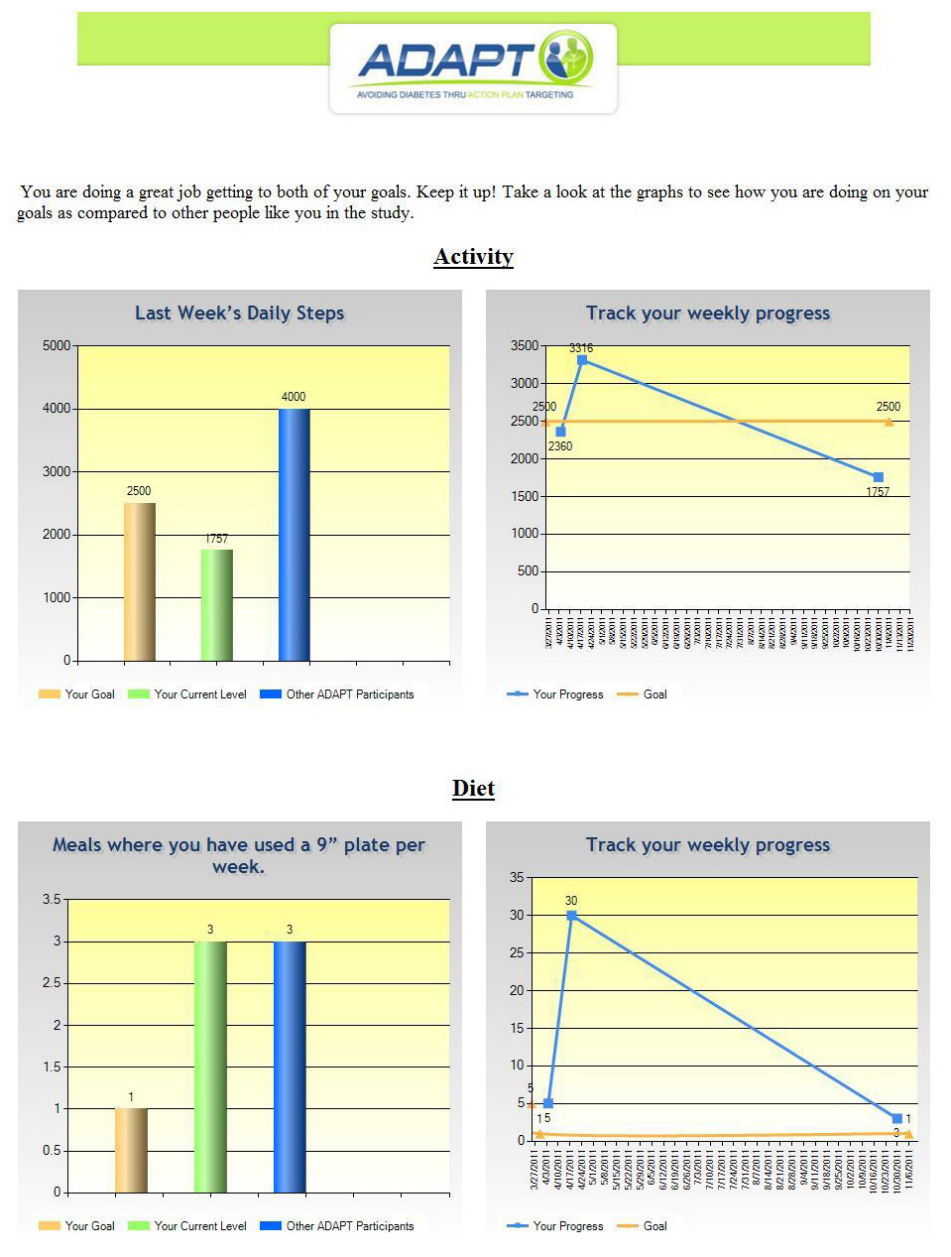
**Screenshot of ADAPT website feedback display**. ADAPT = Avoiding Diabetes Thru Action Plan Targeting.

The RCT consisted of three regular clinical visits at zero, three, and six months (see Figure 5). Patients in the control group simply had regular visits with their providers and were given a brochure about prediabetes before each visit. For patients in the intervention group, prior to each visit, the brief goals-elicitation questionnaire was administered. These data were then entered into the EHR so that the baseline level of the activity and diet goal was displayed to the provider. During the visit, the ADAPT tool was launched (if selected by the provider), and the action plans were negotiated between patient and provider. The providers had the option of using the behavior-change prop of their choice, and afterwards, the patient was given the behavior-change sample. Between each visit with their provider, patients in the intervention group received an email with a web link for an implementation-intention exercise two days after the visit, weekly behavior change tips, and biweekly web forms to record their average steps per day and self-reported level of their diet goal. Once patients have entered their data about their number of steps and diet behaviors, they see a screen displaying their progress over time and in relation to other study participants (see Figure 6). These activities are repeated at each visit, and after six months, both control and intervention patients are mailed home a pedometer to wear for another one week, after which they return it as well as complete a closeout survey.

### Measures

#### Baseline

##### Patient level

Patient characteristics, including age, gender, body mass index, hemoglobin A1C, lipid levels, and medications are captured via EHR chart review. A baseline patient survey based on validated survey instruments assesses the following domains: demographics, medical history, health status [31], physical activity [32], diet [33], family history, prediabetes knowledge, diabetes risk perception [34], weight history [32], social support [32], depression [35], anxiety [36], stage of change [16], and locus of control [37].

##### Provider level

Provider characteristics, including age, gender, years of practice, and attitudes towards prediabetes counseling, are captured via self-report.

### Follow-up

#### Patient level

##### Three month

Intervention and control patients will have fasting preserved glucose and hemoglobin A1C measured.

##### Six month

Intervention and control patients complete a slightly modified version of the baseline survey after their six-month visit (excluding static components such as demographics). Intervention patients also complete a brief survey on their impression of the ADAPT tool components. The pedometer is then mailed home again, and the patients are instructed to wear it for another week and then mail it back to the study team.

#### Provider level

##### Six month

Intervention and control providers are again surveyed about their attitudes towards prediabetes counseling. Intervention providers only are also surveyed about their impression of the ADAPT tool components.

##### Process outcomes

The process outcomes are designed to assess the utilization of the ADAPT tool components by providers and patients. This is a critical outcome because poor provider utilization of clinical decision support and other evidence-based medicine and quality improvement tools have been a frequent barrier to their success [38]. Measured markers of utilization include rate of accepting the ADAPT tool in eligible patients, using the goal-setting flowsheet, and use of the bundled order set linked to the goal-setting activity.

##### Outcome

The outcome measurements are designed to detect changes in patient behaviors that are most likely to result from use of the ADAPT tool. The primary outcome is the difference between intervention and control patients in the change in mean steps per day at baseline and after six months (a difference of differences). Secondary outcomes include the six-month difference of differences in hemoglobin A1C and self-reported diet between the two groups.

##### Data monitoring and quality control

Weekly reports are generated to track the frequency of the tool triggering, including the use of each component of the ADAPT EHR components and the website. Periodic chart reviews are conducted to monitor the appropriateness of tool triggering and to investigate any concerns raised by providers regarding usability or workflow disruptions.

### Statistical analysis

The primary outcome for this pilot randomized control trial will be the change in physical activity as measured in steps/day at six months among prediabetes patients randomized to providers using the ADAPT system compared to those seeing providers in the control arm. We will use descriptive statistics to summarize the variables and detect outliers, data entry mistakes, and missing values. Intervention- and control-group differences in physical activity changes (pre - post) will be tested using a t-test or Wilcoxon test as appropriate, depending on the normality of the data. To further test the effect of ADAPT, we will use a generalized estimating equation model with clinician as the cluster variable, mean difference in steps/day as the outcome variable, and intervention group as the only explanatory variable. Given the nature of the possible relationship between patients in a cluster, we will use an exchangeable correlation structure for parameter estimation. Secondary analysis will use the same methods to examine differences in hemoglobin A1C and diet between those receiving the intervention versus control. Additional analyses accounting for baseline variables will be performed using generalized linear models. If appropriate, missing data will be accounted for using multiple imputation methods.

As this is a pilot RCT, it is underpowered to detect anything but an extremely large change in the primary outcome of physical activity. Based on pilot resources, we designed the pilot trial with a sample size of 60 patients (30 intervention and 30 control) as this provides us 49% power to detect a difference of the recommended physical activity change goal (2000 steps; standard deviation = 3649) [39,40].

## Discussion

While there is consensus that primary care providers need to be part of the battle against the rising epidemic of obesity and diabetes, their effectiveness in counseling for behavior change to date has been limited. Increasing the persuasive power of primary care providers in the provider-patient relationship may help turn the tide against diabetes. The ADAPT study incorporates persuasive design elements grounded in behavior-change theory and delivers them at the point of care in a workflow-friendly manner. The prototype tool is the product of a unique multidisciplinary collaboration and utilized innovative usability methods to create a system that is feasible for daily use in primary care. In addition, it uses web-based platforms to extend the relationship between provider and patient without adding additional work to the provider. It also utilizes each stage of the provider-patient encounter, including the previsit and postvisit period, to embed and synergize persuasive methodologies to help boost the efficacy of provider counseling and help patients change their lifestyle behaviors to prevent the onset of diabetes. Moreover, the ADAPT tool has significant potential to be widely and easily disseminated among primary care providers with an EHR system.

## Competing interests

The authors declare that they have no competing interests.

## Authors' contributions

DMM and JLL conceived the study concept, protocol, and design; supervised implementation and coordination; conducted analyses; and drafted the manuscript. All authors read and approved the final manuscript.
